# AI-based algorithms trained with real-world data for suicide risk prevention in adult mental health patients: The IDICIUS Project

**DOI:** 10.1192/j.eurpsy.2026.12068

**Published:** 2026-07-31

**Authors:** M. Fradera, C. Peña-Gómez, X. Sánchez Corrales, M. Caravaca, D. Roche, C. Pontes, E. Barberia, J. Giraldo, D. Palao

**Affiliations:** 1 Research Support Unit, Institut d’Investigació i Innovació Parc Taulí (I3PT-CERCA); 2 Unitat Mixta de Neurociències Traslacional I3PT-CERCA-INc-UAB, Universitat Autònoma de Barcelona, Sabadell; 3 Centro de Investigación Biomédica en Red de Salud Mental (CIBERSAM), Instituto de Salud Carlos III, Madrid; 4 Institut d’Investigació i Innovació Parc Taulí (I3PT-CERCA), Sabadell; 5 Research Institute for Evaluation and Public Policies (IRAPP), Universitat Internacional de Catalunya (UIC); 6 Digitalization for the Sustainability of the Healthcare System (DS3); 7 Departament de Farmacologia, de Toxicologia i de Terapèutica, Universitat Autònoma de Barcelona; 8 Servei de Farmacologia Clínica, Hospital de la Santa Creu i de Sant Pau, Barcelona; 9 School of Medicine and Health Sciences, Forensic Pathology Department. Institut de Medicina Legal i Ciències Forenses de Catalunya (IMLCFC), Reus; 10 Laboratory of Molecular Neuropharmacology and Bioinformatics, Unitat de Bioestadística and Institut de Neurociències, Universitat Autònoma de Barcelona, Bellaterra; 11 Department of Mental Health, Institut d’Investigació i Innovació Parc Taulí (I3PT-CERCA), Sabadell; 12 Department of Psychiatry and Forensic Medicine, Medical School, Universitat Autònoma de Barcelona, Bellaterra, Spain

## Abstract

**Introduction:**

Suicidal behaviour is a major public health challenge, characterized by heterogeneity and limited predictability in clinical practice. Approximately 50-80% of suicide victims had prior healthcare contact, with 60% within the previous month. Electronic health records (EHRs) provide longitudinal data that, when analysed with artificial intelligence (AI), can uncover complex patterns preceding suicidal behaviour. The IDICIUS project aims to translate this potential into a hospital-based early warning system to support clinical decision-making.

**Objectives:**

To develop machine learning models for suicide risk prediction using real-world data from EHRs, creating a hospital-based early warning system to complement clinical assessment.

**Methods:**

We conducted a retrospective, population-based study including 41,557 adult patients followed in Parc Taulí Mental Health Service between January 1, 2018, and June 1, 2024. Four RWD sources were integrated: mental health EHRs, Catalonia Suicide Risk Code registry, forensic suicide death records, and outpatient medication data. The final dataset comprised 112 variables and 32,661 adult patients (age range 18–95 years, mean 47.08±19.39, 55.9% female), of whom 2,764 exhibited suicidal behaviour (suicide attempt, active ideation, or death). Models were trained using multiple machine learning algorithms, including logistic regression, support vector machines, ensemble methods (Gradient Boosting, XGBoost, CatBoost, Random Forest, AdaBoost), and deep learning. Performance was assessed on a separate test set using ROC-AUC, precision, recall, accuracy, and F1-score. Hyperparameters were tuned with cross-validation, prioritising recall to minimise false negatives. To address class imbalance, under-sampling was applied.

**Results:**

As shown in Image 1, ensemble methods outperformed traditional statistical models. Gradient Boosting and XGBoost achieved the best overall performance with ROC-AUC >0.95, accuracy >0.91, recall 0.84–0.85, and F1-scores around 0.68. While precision was moderate (0.51–0.58), high recall ensured that most true positive cases were captured. Deep learning models showed acceptable performance (ROC-AUC 0.90, recall 0.69), but did not surpass ensemble methods. Logistic regression and elastic net yielded lower ROC-AUC (<0.85) and F1-scores (<0.40). The most important features of the two best-performing models are presented in Images 2 and 3.

**Image 1:**
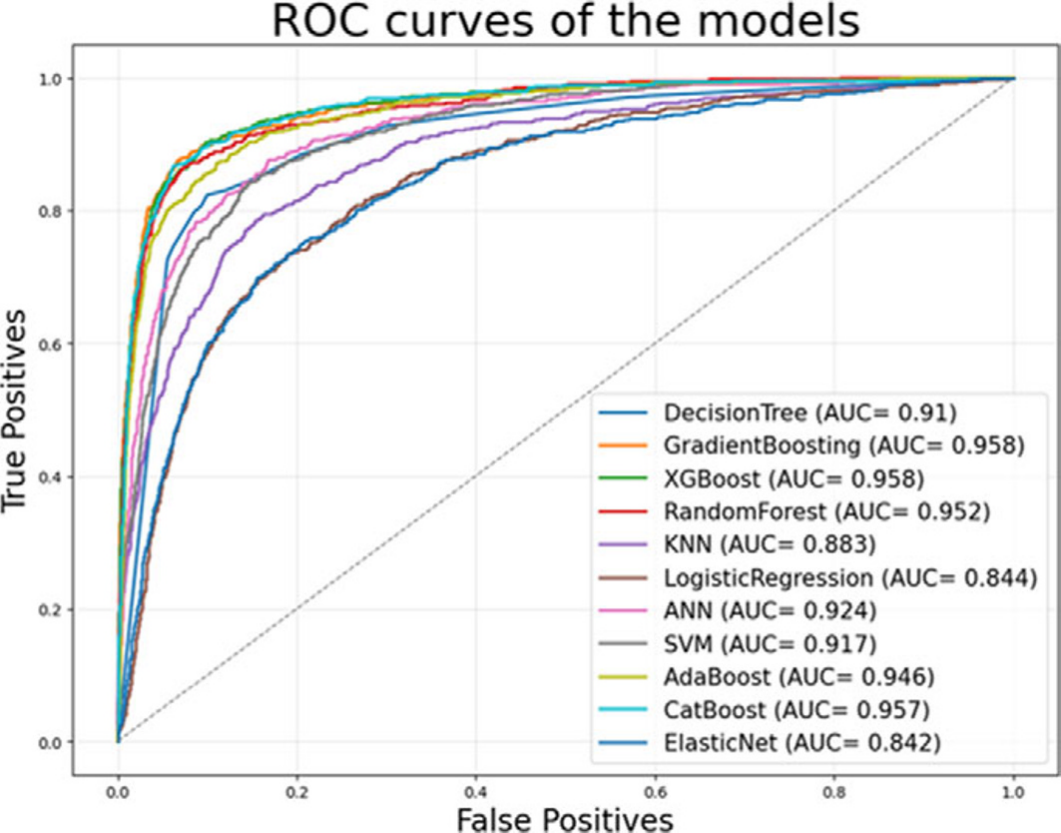
Image 1: Long description.

**Image 2:**
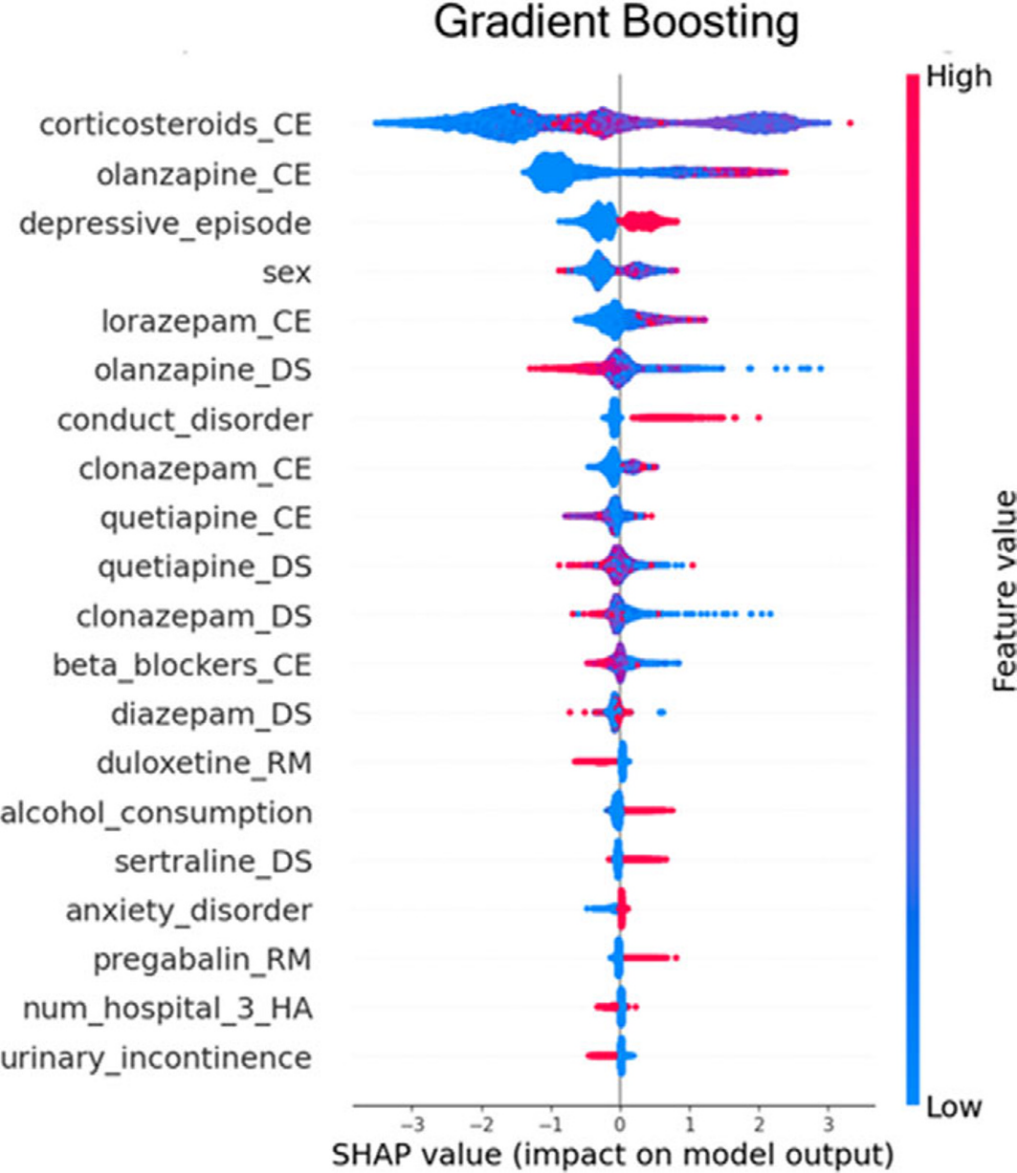
Image 2: Long description.

**Image 3:**
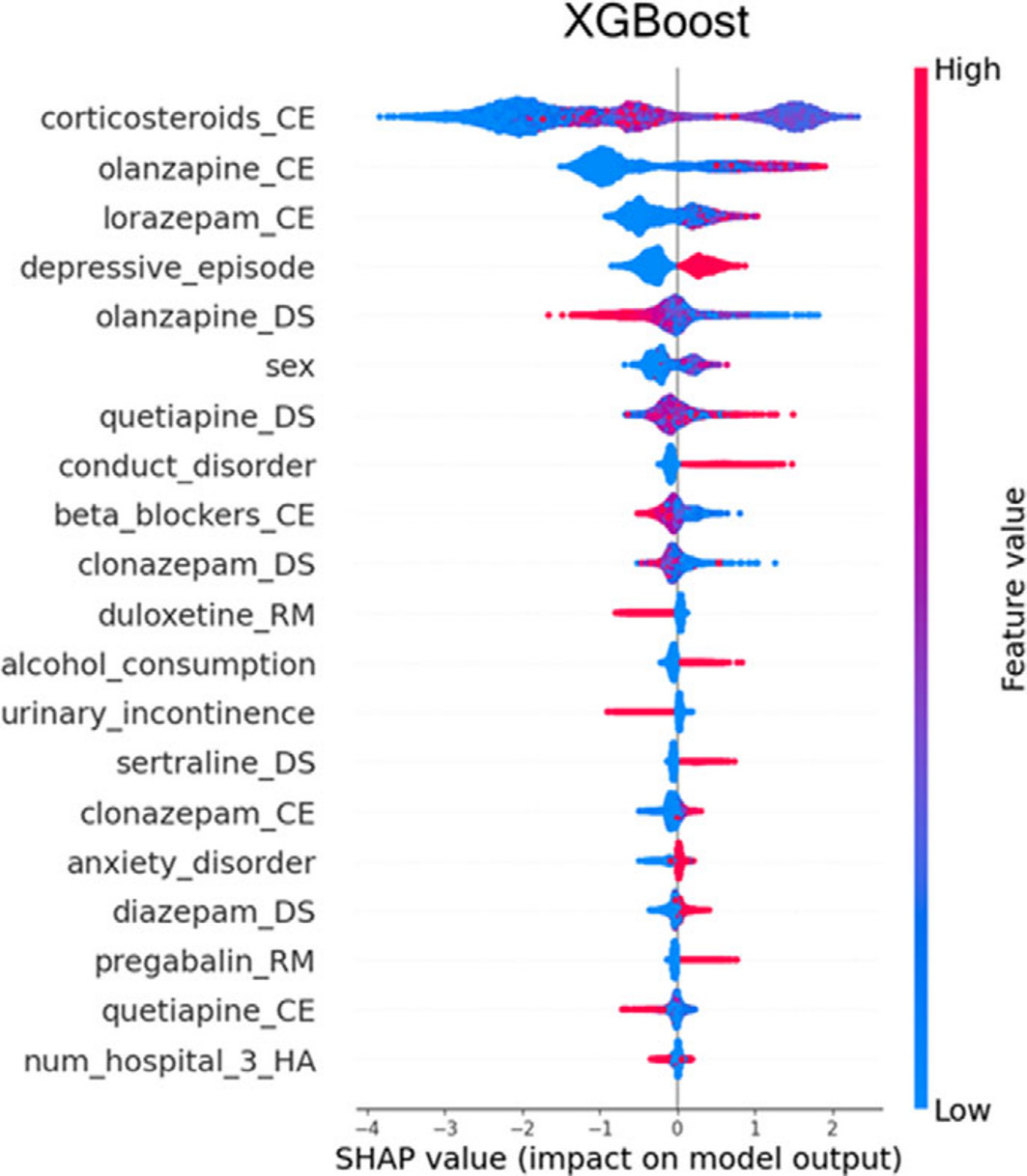
Image 3: Long description.

**Conclusions:**

Integrating multiple RWD sources enabled the development of robust AI-based models for suicide risk prediction. Ensemble methods, particularly Gradient Boosting and XGBoost, demonstrated the best trade-off between recall and precision, supporting their use in early warning systems. Future steps include prospective clinical validation and large-scale implementation in hospital EHRs to assess real-world impact on suicide prevention.

**Disclosure of Interest:**

None Declared

## Long descriptions

### Image 1: Long description

The graph is titled R O C curves of the models. The x-axis is labeled False Positives and the y-axis is labeled True Positives, both ranging from 0.0 to 1.0. A dashed gray diagonal line represents a random classifier baseline. Eleven colored curves originate at 0,0 and arc toward the top-left corner 0,1 before ending at 1,1.

Models are listed in the legend with their A U C values.

* GradientBoosting (orange), XGBoost (green), and CatBoost (cyan) show the highest performance with A U C values of 0.958, 0.958, and 0.957 respectively, following the steepest curves toward the top-left.

* RandomForest (red) has an A U C of 0.952.

* AdaBoost (yellow-green) has an A U C of 0.946.

* A N N (pink) has an A U C of 0.924.

* S V M (gray) has an A U C of 0.917.

* DecisionTree (blue) has an A U C of 0.91.

* K N N (purple) has an A U C of 0.883.

* LogisticRegression (brown) has an A U C of 0.844.

* ElasticNet (light blue) has the lowest performance with an A U C of 0.842, following the curve closest to the diagonal baseline.

Navigate back to Image 1:.

### Image 2: Long description

A S H A P summary plot titled Gradient Boosting. The x-axis represents the S H A P value (impact on model output) ranging from negative 3 to positive 3, with a vertical baseline at 0. The y-axis lists 20 features from top to bottom in descending order of importance.

On the right, a vertical color bar legend labeled Feature value ranges from Low (blue) at the bottom to High (pinkish-red) at the top.

Key feature distributions include:

* corticosteroids_C E: The most important feature, showing a wide distribution where high feature values (red) correlate with negative S H A P values and low feature values (blue) spread across both negative and positive S H A P values.

* olanzapine_C E: High values (red) are concentrated on the positive side of the baseline.

* depressive_episode: High values (red) show a positive impact on the model output.

* sex: Low values (blue) are concentrated slightly to the left of zero, while high values (red) are slightly to the right.

* olanzapine_D S: High values (red) are concentrated on the negative side, while low values (blue) extend far into the positive side.

* conduct_disorder: High values (red) show a strong positive impact.

* urinary_incontinence: The least important feature listed, with high values (red) showing a slight negative impact.

Navigate back to Image 2:.

### Image 3: Long description

A dot plot titled XGBoost. The x-axis is labeled S H A P value (impact on model output) with a scale from negative 4 to positive 2. The y-axis lists 20 features from top to bottom.

1. corticosteroids underscore C E

2. olanzapine underscore C E

3. lorazepam underscore C E

4. depressive underscore episode

5. olanzapine underscore D S

6. sex

7. quetiapine underscore D S

8. conduct underscore disorder

9. beta underscore blockers underscore C E

10. clonazepam underscore D S

11. duloxetine underscore R M

12. alcohol underscore consumption

13. urinary underscore incontinence

14. sertraline underscore D S

15. clonazepam underscore C E

16. anxiety underscore disorder

17. diazepam underscore D S

18. pregabalin underscore R M

19. quetiapine underscore C E

20. num underscore hospital underscore 3 underscore H A

A vertical gray line at S H A P value 0 represents the baseline. To the right of the plot is a vertical color bar legend labeled Feature value, ranging from Low in blue at the bottom to High in red at the top.

Data distribution highlights:

* corticosteroids underscore C E shows a wide distribution of blue dots (low feature value) extending to negative 4, indicating a strong negative impact on the model output.

* depressive underscore episode shows red dots (high feature value) clustered on the positive side of the x-axis.

* olanzapine underscore D S shows red dots extending far to the left (negative impact) and blue dots extending to the right (positive impact).

Navigate back to Image 3:.

